# Application of embolization microspheres in interventional therapy of malignant non-hypervascular tumor of liver

**DOI:** 10.18632/oncotarget.16286

**Published:** 2017-03-16

**Authors:** Huanzhang Niu, Tingwei Du, Quanping Xiao, Xin Hu, Dongmin Li, Chao Wang, Wanqin Gao, Taohong Xing, Xiangmei Xu

**Affiliations:** ^1^ Department of Interventional Radiology, The First Affiliated Hospital, and College of Clinical Medicine of Henan University of Science and Technology, Luoyang, China

**Keywords:** malignant liver tumors, transarterial embolization, non-hypervascular, microspheres

## Abstract

**Objective:**

To investigate the efficacy and safety of transarterial embolization (TAE) using embolization microspheres in the treatment of non-hypervascular malignant liver tumors.

**Methods:**

Patients with malignant non-hypervascular liver tumors, who were treated with TAE using embolization microspheres, were selected and analyzed retrospectively. The technical success rate, tumor response, and complications were assessed.

**Results:**

Six patients were included in the study: 1 patient each with hepatocellular-cholangiocarcinoma, intrahepatic cholangiocarcinoma, hepatic metastasis after resection of common bile duct carcinoma, liver metastasis from colon cancer, liver metastasis from esophageal cancer, and liver metastasis from pancreatic cancer. The technical success rate was 100%. At 1 and 3 months after TAE, tumor local reactions were seen in 6/6 and 2/6 patients, respectively, and the tumor necrosis rates were 48%-73% and 22%-68%, respectively. The main complications were those related to the embolization syndrome, including 1 case of liver abscess and 1 case of severe pain on the first day after embolization.

**Conclusion:**

TAE with embolization microspheres is safe and effective in non-hypervascular liver tumors. It is a feasible option for palliative therapy of these tumors.

## INTRODUCTION

Malignant tumor of the liver, which includes primary and secondary liver cancers, are a relatively common form of malignancy. A considerable proportion of patients have inoperable disease and require minimally invasive treatments, such as transarterial chemoembolization (TACE), transarterial radioembolization (TARE), radiofrequency ablation, microwave ablation, cryoablation, chemical ablation, and targeting therapy (e.g., oral sorafenib), or some combination of them [1–6]. However, the fact that so many methods exist is indicates that no one is good enough for application in all patients; each treatment modality has its indications. For example, local ablation treatment is mainly applied in solid tumors of relatively small size (<5 cm diameter), whereas transarterial embolization (TAE) is preferred for solid tumors with rich blood supply. Since most of primary hepatocellular carcinoma have rich blood supply, embolization is effective in large and multiple hepatocellular carcinomas. TAE or TACE has been widely used in the treatment of a variety of hypervascular tumors, and has displayed significant therapeutic efficacy [7]. However, TAE is not so effective in tumors without rich blood supply due to limitations of the embolization materials. This problem has not received much attention, and there are no relevant reports in literature.

In recent years, calibrated embolization microspheres (trimethylol acrylic acid microspheres; TGMs), with spherical shape, smooth surface, and uniform size distribution, have been used for embolization and has shown good results [8, 9]. Compared to the gelatin sponge particles and polyvinyl alcohol (PVA) particles, they do not tend to aggregate in the proximal part of the blood vessel, and it is possible to predict which level of blood vessels will be embolized [10].

This retrospectively study was designed to examine the therapeutic efficacy of TAE using TGMs in large liver tumors with poor blood supply.

## MATERIALS AND METHODS

### Patients

**Figure 1:**
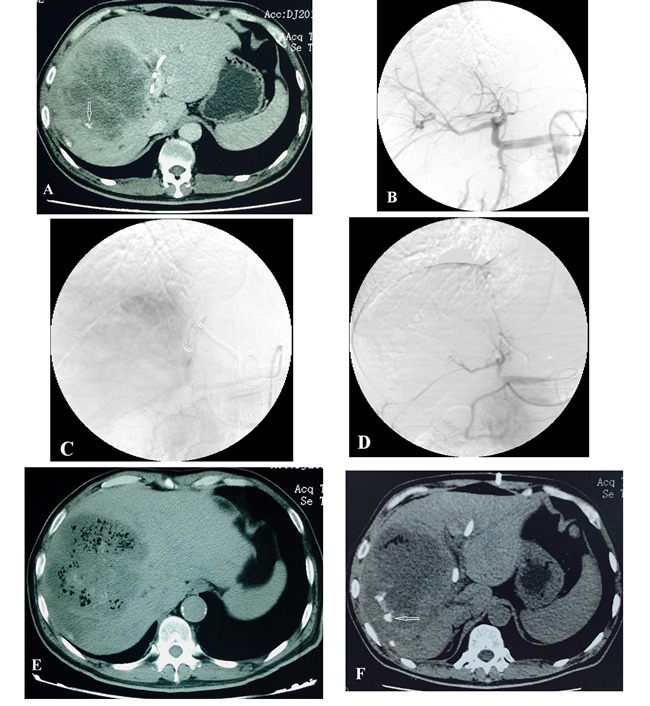
Male patient, 54 years old, with hepatocellular cholangiocarcinoma CT scan shows a hypovascular tumor. The patient had undergone PTCD and TACE (with 1 week interval) 1 month earlier. At review, there was increase in the size of the tumor. Microspheres TAE therapy was applied immediately. **A**. CT scan shows lesions located in the right lobe of the liver before TAE, poor blood supply, and a small amount of lipiodol deposited around the edge of lesion (white arrow). **B**. **C**. Hepatic artery angiography before embolization, shows that the blood supply is not rich. **D**. After embolization, the tumor blood vessels have disappeared, and the main and secondary branches of the hepatic artery are visible. **E**. CT plain scan 1 week after embolization shows visible tumor necrosis; many bubbles can be seen. **F**. Three months after embolization, most lesions have undergone necrosis; a small amount of gas and a little lipiodol deposition can be seen at the edges of the lesions (white arrow).

All patients were selected from among the patients attending the Interventional Therapy Department of our hospital between August 1, 2015, and February 29, 2016. Patients were eligible for inclusion if they had 1) a confirmed non-hypervascular malignant tumor of liver, and were unwilling or unsuitable for surgery; 2) TMGs only was applied during the embolization, no other embolic agents such as iodized oil, gelatin sponge and so on. Patients with poor Life expectancy (<3 months) were excluded, such as liver tumor rupture and bleeding. Definition of non-hypervascular malignant tumor of liver: Two senior imaging physicians unanimously determined that intrahepatic tumors showed no enhancement or mild enhancement in the arterial phase of CT enhancement (The average CT value of arterial phase increased less than 20 Hu than plain scan in the lesion) [Figure 1A].

All investigations were conducted according to the principles expressed in the Declaration of Helsinki. The study was approved by the Ethics Committee of our hospital.

### Embolization technique and treatment

We followed a standard treatment procedure. First, angiography of the common hepatic artery was performed to identify the feeding arteries [Figure 1B, 1C], including unusual feeding vessels such as the inferior phrenic artery, superior mesenteric artery, and so on. We used a microcatheter to superselectively target the feeding artery and then slowly injected the microspheres for embolization. In view of the relatively sparse blood supply and the small blood vessels in non-hypervascular tumors, only 2 sizes of microspheres (Embosphere; Merit Medical Systems Inc., South Jordan, UT, USA) were used in this study, namely, 100-300 μm and 300-500 μm. After the microcatheter reached the target blood vessel, the microspheres suspension was prepared [11]: 1 mL microspheres was added into a 15 mL mixture of physiological saline solution and iodinated contrast agent (in a ratio of 1:1). This was drawn into a 20 mL syringe (which functioned as a storage syringe). A 1 mL syringe (which functioned as the injection syringe) was then connected to the 20 mL syringe via a three-way stopcock, and the suspension was mixed by repeated suction and expulsion. The microcatheter was then connected, and the microspheres suspension was injected slowly into the target vessel. As a cost-saving measure, the 100-300 μm microspheres were injected first, and microspheres of larger size were used only if the embolization was not sufficient. When fluoroscopy demonstrated slowing of blood flow, angiography was performed to confirm that the tumor vessels had disappeared, after which the procedure was terminated [Figure 1D]. If the tumor vasculature reappeared 5 minutes after the embolization, the procedure was repeated.

### Treatment before and after the procedure

A second-generation cephalosporin antibiotic was administered 30 minutes before the procedure, with another dose being given after the operation. Additionally, supportive and symptomatic treatment was provided to all patients as needed.

### Evaluation and analysis

Technical success of TAE was defined as: 1) successful catheterization of the target vessel; and 2) disappearance of >90% tumor blood vessels demonstrated by postprocedure angiography (this was decided by consensus between two interventional physicians above the rank of deputy chief physician). Enhanced CT was performed before the procedure, 1 month after the procedure, and 3 months after procedure to assess the change in tumor size and the rate of tumor necrosis. Objective evaluation of efficiency of tumor therapy was according to the RECIST 1.1 criteria [12].

## RESULTS

In all, 6 patients (4 men and 2 women) were selected in this study; the average age was 64.67 years (range, 53-80 years). The characteristics of the patients are shown in Table 1. Of the 6 patients, 3 had previously received TACE, but the results were unsatisfactory; 1 had undergone resection of the head of the pancreas and the duodenum, and received biological treatment, focused ultrasound, TACE, and so on; and 2 had undergone percutaneous transhepatic biliary drainage (PTBD).

**Table 1:** Basic characteristics of the 6 patients in the study

Tumor pathological type	Sex	Age (year)	Amount of embolization vascular	Number of liver tumors	Lesion dimentions (mm)
Hepatocellular cholangiocarcinoma	Male	53	3	1	142 × 128 × 121
Intrahepatic cholangiocarcinoma	Male	65	2	1	168 × 142 × 127
Common bile duct carcinoma	Female	61	3	3	81 × 68 × 51 46 × 43 × 41 32 × 30 × 28
Carcinoma of colon	Male	67	3	2	101 × 94 × 89 57 × 55 × 46
Esophageal cancer	Female	80	3	1	112 × 104 × 98
Pancreatic cancer	Male	62	4	2	97 × 90 × 79 66 × 62 × 56
Total			18	10	

The technical success rate in this study was 100%. Totally, 18 targeting vessels were embolized in the 6 patients; among these, there was 1 patient with anomalous origin of the right hepatic artery from the superior mesenteric artery. In 4 patients, anticancer drugs (cisplatin, 5-fluorouracil, and gemcitabine) were administered via local hepatic artery infusion. Microspheres of two sizes (100-300 μm and 300-500 μm) were used in all procedures. The details are shown in Table 2.

**Table 2:** Amount of microspheres used for embolization

Tumor pathological type	Number of liver tumors	Amount of microspheres used (mL)	
		100-300 μm	300-500 μm
Hepatocellular cholangiocarcinoma	1	3	0
Intrahepatic cholangiocarcinoma	1	2	1.2
Common bile duct carcinoma	3	2.7	0
Carcinoma of colon	2	2	0.5
Esophageal cancer	1	2	1.3
Pancreatic cancer	2	2.5	0

Note: Dosage (mL) is the volume of microspheres; the actual dosage is the volume of microspheres multiplied by the dilution ratio.

The local reactions of all patients after TAE are shown in Table 3. In the 6 patients, 0 case of complete response (CR) and 6 cases of partial response (PR) after TAE were observed, the tumor necrosis rate reached 48%-73%. Three months after TAE, there was no case of CR, 1 case of SD, 2 cases of PR, and 3 cases of PD; the tumor necrosis rate was 22%-68%. In two of the larger lesions (maximum diameter >10 cm), large areas of necrosis associated with bubbles were observed on CT scan 1 week after TAE [Figure 1E, 1F].

**Table 3:** Response of lesions in the 6 patients

Tumor pathological type	*n*	At 1 month						At 3 months					
		*n*	CR	PR	SD	PD	NR	*n*	CR	PR	SD	PD	NR
Hepatocellular cholangiocarcinoma	1	1	0	1	0	0	60%	1	0	1	0	0	64%
Intrahepatic cholangiocarcinoma	1	1	0	1	0	0	55%	1	0	0	0	1	30%
Common bile duct carcinoma	3	3	0	3	0	0	73%	3	0	3	0	0	68%
Carcinoma of colon	2	2	0	2	0	0	62%	4	0	1	0	1	22%
Esophageal cancer	1	1	0	1	0	0	61%	1	0	0	1	0	60%
Pancreatic cancer	2	2	0	2	0	0	48%	3	0	0	2	1	39%
Total	10	10	0	10	0	0		13	0	5	3	3	

Abbreviations: CR = complete response, PR = partial response, SD = stable disease, PD = progressive disease, NR = necrosis rate

Within 1 week after TAE, 2 patients with massive tumors reported relief in abdominal pain; the pain soon disappeared and analgesics could be discontinued. There were no surgery-related deaths. However, the 2 patients who had undergone PTCD died during the follow-up period: one due to gastrointestinal bleeding 4 months after TAE, and the other due to cancer metastasis accompanied by multiple organ failure induced by biliary tract infection 5 months after TAE.

Postoperative complications were mainly related to the postembolization syndrome, and included pain, nausea, vomiting, fever, and elevated blood pressure. These symptoms generally disappeared within 7 days. In patients with relatively larger lesions, the fever lasted for more than 2 weeks, and the younger patients with large lesions had severe pain response. Five days after TAE, the serum bilirubin level of 1 patient who had undergone prior PTCD increased sharply, but it had decreased obviously at review 5 days later. The most serious complication encountered was liver abscess, which was seen in 1 patient; this patient was discharged from hospital after treatment with drainage of the abscess and appropriate antibiotics.

## DISCUSSION

This study aimed to study the efficacy of TAE with a new type of embolization material—the TGM—in the treatment of non-hypervascular malignant tumors of the liver. Good short-term effects were achieved after embolization: the tumor necrosis rate was high, symptoms were relieved, and the quality of life was improved. The main complications were those related to the postembolization syndrome. We believe that this form of TAE can be an option for palliative treatment of large non-hypervascular tumors of the liver.

In 1996, Laurent et al. [13] reported the development of TGMs and described their in vitro characteristics. TGMs are made of nonabsorbable cross-linked acrylic polymer and embedded gelatin. These microspheres are spherical particles after calibration, with uniform diameter, soft (compressible) consistency, and a hydrophilic smooth surface. They are easily injected through microcatheters, and achieve a uniform distribution in the vascular networks of the target organ [10]. The study showed that [14, 15], according to the diameter of vessels in lesions, microspheres with appropriate diameter were selected. With the guidance of blood flow, the microspheres could stay in the corresponding blood vessels, and achieve the goal of targeted embolization. Pathologic examination has shown that the microspheres are mainly distributed in the tumor vessels, and the diameter of the microspheres was consistent with the diameter of the blocking vessels.

The microsphere has been approved for use in a variety of benign and malignant tumors with rich blood supply and has been widely applied clinically [16, 17]. In clinical practice, lipoidol has not proved to be effective for embolization of poorly vascularized tumors or secondary liver tumors; it is cleared early and the treatment effects have been unsatisfactory. Due to these reasons, we decided to apply TGMs in the interventional treatment of non-hypervascular liver tumors.

In liver tumors with rich blood supply, tumor staining is obvious when hepatic artery angiography is performed; the feeding artery is enlarged, and the target blood vessels are easily identified and catheterized. Embolization agents easily enter into tumor vessels because the pressure in tumor vessels is usually lower than that in surrounding normal liver tissues. Non-hypervascular liver tumors, however, do not display these characteristics due to the presence of larger amounts of fibrous tissue and other interstitial components and the sparse vascular network. Therefore, in these tumors, before embolization, contrast must be injected at or near the lesions by the microcatheter for identifying the feeding artery(uneven thickness, tortuous and vascular wall stiffness, less smooth and so on), CT or MRI could be used to identify the feeding artery too. Superselective embolization was performed to avoid large quantities of embolic agent from entering into normal liver tissue. The use of a DSA machine with CT C-arm function can help accurately identify the artery supplying the lesion and thus make the procedure much easier. After embolization by microspheres, pathologic examination of tumors with rich blood supply has shown that most of the microspheres were distributed in tumor blood vessels, and that the blood flow in the hepatic artery was well maintained [18].

In this study, after superselective microcatheter insertion, the majority of the lesions were treated by a slow embolization. Necrosis of most of the lesions was achieved, without obvious necrosis of normal liver tissue or significant deterioration of liver function, indicating that most of the embolization microspheres had entered into tumor tissue. To ensure that the embolization only targets the tumor, the microspheres should be diluted maximally and injected slowly, with about 1 mL of the suspension being injected over >1 minute.

In this study, different degrees of necrosis were seen; larger lesions decreased more in size, indicating greater degree of necrosis (up to 73%). All patients, however, benefited from the procedure, with 100% (6/6) of patients having PR at 1 month after embolization. At 3 months after embolization, 33.33% (2/6) had PR and 16.67% (1/6) had SD. Following embolization, tumor volume was markedly reduced, symptoms such as epigastric pain decreased and even disappeared, and the patients’ quality of life was greatly improved. However, we do not have statistics on survival. A sharp rise in serum bilirubin was seen in one patient on the 5^th^ day after embolization, which was likely due to compression of a narrow biliary tract by tumor edema. We did not come across any case of serious infection, probably because we took the precaution of administering prophylactic antibiotics to patients with large tumors and biliary tract disease.

The microsphere size for use in any particular tumor is not specified; it is selected on the basis of the radiographic findings [19]. In our study, two sizes of microspheres (100-300 μm and 300-500 μm) were used. When the tumor blood supply is not abundant, the few blood vessels that are present have smaller lumens than the vessels of tumors with rich blood supply. We therefore did not have to use microspheres of larger diameters. CT was applied to exclude patients with arteriovenous shunt before surgery, however, it was impossible to avoid the de novo appearance of such shunts, and so we did not use smaller microspheres, meanwhile with the consideration of bile duct injures. Although, theoretically, any patient who can be embolized with lipiodol can also be embolized with microspheres of >40 μm, the metabolic degradation characteristics of the two in vivo are not the same.

This study has some limitations. First, the sample size is small, and the proportion of huge liver tumors was high. Second, the follow-up was for a short duration. All patients did not receive a second embolization. because of seriously ill and bad prognosis; they were only seeking immediate relief of symptoms. A third limitation is that some of the patients in this group also received chemotherapy drugs during the procedure, and so the complications might not be entirely attributable to the TAE.

## CONCLUSIONS

TAE using TGMs is a feasible and safe option for palliative treatment of liver tumors, especially huge, poorly vascularized tumors.
